# Medical Image Segmentation Algorithm for Three-Dimensional Multimodal Using Deep Reinforcement Learning and Big Data Analytics

**DOI:** 10.3389/fpubh.2022.879639

**Published:** 2022-04-08

**Authors:** Weiwei Gao, Xiaofeng Li, Yanwei Wang, Yingjie Cai

**Affiliations:** ^1^College of Information and Technology, Wenzhou Business College, Wenzhou, China; ^2^Department of Information Engineering, Heilongjiang International University, Harbin, China; ^3^School of Mechanical Engineering, Harbin Institute of Petroleum, Harbin, China; ^4^The First Psychiatric Hospital of Harbin, Harbin, China

**Keywords:** deep reinforcement learning, three-dimensional multimodal, wavelet shrinkage, medical image segmentation, high-frequency signal component

## Abstract

To avoid the problems of relative overlap and low signal-to-noise ratio (SNR) of segmented three-dimensional (3D) multimodal medical images, which limit the effect of medical image diagnosis, a 3D multimodal medical image segmentation algorithm using reinforcement learning and big data analytics is proposed. Bayesian maximum a posteriori estimation method and improved wavelet threshold function are used to design wavelet shrinkage algorithm to remove high-frequency signal component noise in wavelet domain. The low-frequency signal component is processed by bilateral filtering and the inverse wavelet transform is used to denoise the 3D multimodal medical image. An end-to-end DRD U-Net model based on deep reinforcement learning is constructed. The feature extraction capacity of denoised image segmentation is increased by changing the convolution layer in the traditional reinforcement learning model to the residual module and introducing the multiscale context feature extraction module. The 3D multimodal medical image segmentation is done using the reward and punishment mechanism in the deep learning reinforcement algorithm. In order to verify the effectiveness of 3D multimodal medical image segmentation algorithm, the LIDC-IDRI data set, the SCR data set, and the DeepLesion data set are selected as the experimental data set of this article. The results demonstrate that the algorithm's segmentation effect is effective. When the number of iterations is increased to 250, the structural similarity reaches 98%, the SNR is always maintained between 55 and 60 dB, the training loss is modest, relative overlap and accuracy all exceed 95%, and the overall segmentation performance is superior. Readers will understand how deep reinforcement learning and big data analytics test the effectiveness of 3D multimodal medical image segmentation algorithm.

## Introduction

In current medical practice, medical images such as MRI image, CT image, and ultrasonic imaging are important diagnostic basis for diagnosing patients' physical conditions. Doctors or researchers analyze the tissues and organs in the human body through the medical image, obtain the relevant information of the tissues and organs in the human body according to the medical image, and carry out treatment planning in combination with medical knowledge. Therefore, medical image has become an indispensable part of disease diagnosis and treatment and quality. Medical image segmentation is not only a prerequisite for computer-aided diagnosis and many medical image applications, but also an important stage of medical image visual analysis (1, 2). Image segmentation of medical image can help to directly obtain the contour of the target by identifying the target of interest. In diagnosis, it is convenient for doctors to obtain the patient's condition information (3). With the development of medicine, medical images have been continuously optimized to gradually form three-dimensional (3D) multimodal medical images, which make medical images clearer and have higher resolution (4, 5). In order to effectively distinguish the pathological region from the normal region in medical image and enable doctors to diagnose and treat more intuitively, the segmentation of 3D multimodal medical image has become the focus of current research. However, in practical application, the factors such as image device, organ position, and shape still hinder the high-precision segmentation of medical image. The traditional image segmentation algorithm is difficult to overcome the influence of many factors and the effect of segmented medical image is poor.

At present, with the continuous improvement of science and technology, deep reinforcement learning technology has been applied in a large number of fields because this technology has high feature extraction ability. Compared with the traditional segmentation algorithm, it has obvious advantages and is widely used in the process of image segmentation (6). Therefore, a 3D multimodal medical image segmentation algorithm using deep reinforcement learning is proposed in this article. The image segmentation is performed by constructing a deep reinforcement learning model. The main contributions of this article are as follows: (1) We combine wavelet denoising with bilateral filtering to realize 3D multimodal medical image denoising. According to the traditional wavelet denoising method, Bayesian maximum a posteriori estimation method is used to realize wavelet shrinkage and combined with bilateral filter to improve the denoising performance of image; (2) The extended convolution is added and two-dimensional convolution is introduced to improve the receptive field and ensure that comprehensive context information can be obtained during segmentation; and (3) In deep reinforcement learning, the residual network is introduced to make the segmentation results more accurate and improve the training speed of deep reinforcement learning. Using the introduced residual structure, the feature extraction performance of the network can be improved.

## Related Works

Many scholars have studied the related problems of medical image segmentation. Literature (7) studied medical image segmentation based on spatial constraints and fuzzy spatial segmentation. By analyzing the image edge position, the realization of spatial constraints and complete medical image segmentation were determined. However, the algorithm is only suitable for fuzzy and uncertain medical image processing. When it is used for 3D multimodal medical image, the segmentation performance of the algorithm is poor. Literature (8) studied the weakly supervised retinal vascular segmentation algorithm and used the hierarchical clustering algorithm to classify the vascular and non-vascular pixels. Moreover, the classification results based on the driving database were used as the basic facts to train the neural network. By calculating and comparing the image feature differences between the target domain data and the source domain data extracted from the network, the data required for training were extended based on semi-supervised clustering to realize retinal vessel segmentation. The algorithm can make pixels highly reliable, but the algorithm can improve the segmentation accuracy of retinal vessels, but its segmentation effect is poor. Literature (9) studied the automatic segmentation of lung tumors in CT images based on multiresolution residual connected feature flow. Incremental and dense multiresolution residual connected networks were used to detect and segment lung tumors by combining the features of multiple image resolutions and feature levels. The algorithm can effectively improve the image dimension in the segmentation process, but the segmentation operation can only be realized according to the feature flow, so the segmentation quality factor is poor. Literature (10) studied image segmentation combining image weighting and transfer learning and used kernel learning as a method to reduce the difference between training and test data, so as to enhance the performance of image segmentation through continuous learning and training. However, the algorithm does not consider the multimodality of 3D image in the segmentation process, so the definition is poor. Literature (11) studied Pulse Coupled Neural Networks (PCNN) medical image segmentation based on multifeature gray wolf optimized bionic algorithm and used image multifeatures to realize segmentation. However, the algorithm has poor structural similarity between training and actual segmentation, cannot completely realize segmentation, and the signal-to-noise ratio (SNR) is low, resulting in more noise points in the image. Literature (12) studied medical image segmentation based on dice score and the Jaccard index and the relationship within the measurement sensitive loss function group from a theoretical point of view. Moreover, the existence of the optimal weighting scheme of weighted cross entropy was questioned to optimize the dice score and the Jaccard index during the test and realize medical image segmentation through dice score and the Jaccard index. This method optimized medical image segmentation to some extent, but it did not denoise the image, so the effect of medical image segmentation is poor. Literature (13) studied medical image segmentation based on context feedback loop and expressed the segmentation problem as a recursive framework by using two systems. The first is the forward system of encoder-decoder convolutional neural network, which predicts the segmentation results from the input image. The predicted forward system probability output is encoded by a context feedback system based on a complete convolution network. Then, the coding feature space of the complete convolution network is integrated back into the feedforward learning process of the forward system. This method uses the context feedback loop based on complete convolution network and the forward system can learn and extract more advanced image features. Moreover, it fixes the previous errors and improves the segmentation accuracy over time. This method can effectively improve the segmentation accuracy, but it does not denoise the collected medical image, so that the segmentation quality factor is low.

In the process of image segmentation by the above methods, the analysis of influencing factors in the process of image segmentation is not comprehensive, resulting in low relative overlap and signal-to-noise ratio of image segmentation 3D multimodal medical image. Therefore, this article studies the 3D multimodal medical image segmentation algorithm based on deep reinforcement learning and uses wavelet algorithm and bilateral filter to denoise the image to make the image clearer. Then, a deep reinforcement learning model is constructed to realize multimodal medical image segmentation by combining residual structure and dilated residual and deeply supervised U-Net (DRD U-Net) convolution model. The performance of the proposed algorithm is verified by experiments.

## Methodology

### Medical Image Denoising of Three-Dimensional Multimodal Based on Wavelet and Bilateral Filtering

Combined with wavelet denoising and bilateral filtering, 3D multimodal medical image denoising is realized. According to the traditional wavelet denoising method, Bayesian maximum a posteriori estimation method is used to realize wavelet shrinkage and combined with bilateral filter to improve the denoising performance of image.

#### Setting of Wavelet Threshold Function

In order to realize the accurate segmentation of medical image segmentation, it is necessary to denoise the image. In this article, the wavelet threshold function is set to provide the prerequisite for image denoising. The calculation formula of wavelet shrinkage threshold function is constructed $T=\sigma_{N}\sqrt{2\log M}$ for analysis.

where *M* refers to the total number of wavelet coefficients in the corresponding wavelet domain. Because the calculation effect of the general threshold function is not perfect, the general threshold function is redesigned in an improved form, as shown in Equation (1).


(1) $$\begin{array}{l} T_{j}=a_{j}\sigma_{N}\sqrt{2\log M} \end{array}$$

where after decomposition, the number of layers with wavelet coefficients is described by *j* = (1, 2, ..., *J*). The maximum number of decomposition layers is *J*, *a*_*j*_ is the adaptive parameter, and the adaptive parameter corresponding to layer *j* is 2^*J*−*j*+1^.

#### Design of Wavelet Shrinkage Algorithm

In order to obtain the threshold function of wavelet shrinkage, according to the constructed wavelet shrinkage threshold function, the wavelet coefficients of noiseless signal are analyzed by generalized Laplace distribution. Moreover, the Bayesian maximum a posteriori estimation method is used to calculate the a posteriori probability and complete the design of wavelet shrinkage algorithm.

Wavelet coefficients of noiseless signals can be analyzed by generalized Laplace distribution $G_{l,k}^{j}$ and calculate its probability distribution through Equation (2).


(2) $$\begin{array}{l} p_{G}\;(g)=\frac{v}{2s\Gamma \left(\frac{1}{v}\right)}\exp\left(-{\left|\frac{g}{s}\right|}^{v}\right),s,v>0 \end{array}$$

where *p*_*G*_ (*g*) refers to the probability distribution of the coefficient, *g* refers to noise-free signal, subscript (*l, k*) is coordinates in wavelet domain, the gamma function is $\Gamma \left(a\right)=\int_{0}^{\infty }x^{a-1}\exp\left(-x\right)\;dx$, and the scale parameter is *s*. If shape parameter *v* = 1, Equation (2) can be changed to Laplace distribution, as shown in Equation (3).


(3) $$\begin{array}{l} p_{G}\;(g)=\frac{1}{2s}\exp\left(-|\frac{g}{s}|\right),s>0 \end{array}$$

The speckle noise $N_{l,k}^{j}$ in the wavelet domain can be calculated by Gaussian distribution, as shown in Equation (4).


(4) $$\begin{array}{l} p_{N}\;(n)=\frac{1}{\sqrt{2\pi \sigma_{N}}}\exp\left(-\frac{n^{2}}{2\sigma_{N}^{2}}\right) \end{array}$$

where *p*_*N*_ (*n*) refers to the probability distribution of the speckle noise and the noise SD in wavelet domain is described by σ_*N*_. In order to obtain the signal estimation in the domain, the Bayesian maximum a posteriori estimation method is used to calculate the a posteriori probability. It is obtained by Equation (5).


(5) $$\begin{array}{l} p_{G|F}\;(g|f)=\frac{1}{p_{F}\left(f\right)}p_{F|G}\;(f|g)\;p_{G}\;(g)=\\ \frac{1}{p_{F}\left(f\right)}p_{N}\;(f-g)\;p_{G}\;(g) \end{array}$$

where *f* refers to the medical image network signal obtained in the initial state and Equation (3) and Equation (4) are introduced into Equation (5) to obtain Equation (6).


(6) $$\begin{array}{l} p_{G|F}\;(g|f)=\frac{1}{p_{F}\left(f\right)}\cdot \frac{1}{2\sqrt{2\pi s\sigma_{N}}}\times \\ \exp\left(\frac{2\sigma_{n}^{2}|g|-s{\left(f-g\right)}^{2}}{2s\sigma_{N}^{2}}\right) \end{array}$$

To calculate the maximum a posteriori probability, set the result of derivation calculation from ln (*p*_*G*|*F*_ (*g*|*f*)) to *g* is 0. It is obtained by Equation (7).


(7) $$\begin{array}{l} ĝ=\text{sgn }\left(f\right)\text{ max }\left(|f|-\frac{\sigma_{N}^{2}}{s},0\right) \end{array}$$

where *g* is estimated to be *ĝ* and set *f* and *g* are of the same number. The threshold function of wavelet shrinkage is obtained through the Equation (7) calculation. Therefore, the wavelet shrinkage algorithm is expressed by Equation (8).


(8) $$\begin{array}{l} ĝ=\{\begin{array}{l} 0\;f\;\leq \;T_{j}\\ \text{sgn }\left(f\right)\text{ max }\left(|f|-\frac{\sigma_{N}^{2}}{s},0\right)\;f\;>\;T_{j} \end{array} \end{array}$$

#### Combination With Bilateral Filter

The traditional wavelet denoising methods retain the unchanged wavelet coefficients in the low-frequency domain and only deal with the threshold of wavelet coefficients in the high-frequency domain, so the denoising effect is not obvious (14, 15). In order to remove the noise spots in the low-frequency domain, this article combines the bilateral filter to filter the wavelet coefficients in the low-frequency domain. The structure of the bilateral filter is represented by Equation (9).


(9) $$\begin{array}{l} h\;(x)=k^{-1}\;(x)\times \int_{\xi \in \Omega \left(x\right)}f\left(\xi \right)\;c\;\left(\xi ,x\right)\;s\;\left(f\left(\xi \right),f\left(x\right)\right)\;d\xi \end{array}$$

where the normalization factor is described by $k\left(x\right)=\int_{\xi \in \Omega \left(x\right)}c\left(\xi ,x\right)s\left(f\left(\xi \right),f\left(x\right)\right)d\xi$ and the window region with the pixel *x* as the center is Ω(*x*). A bilateral filter is formed by combining two filter cores (16, 17), i.e., the regional kernel *c* (ξ, *x*) and range kernel *s* (*f* (ξ), *f* (*x*)). In the above formula, the distance function from the edge pixel ξ in region Ω(*x*) and the pixel of the central region *x* is *c* (ξ, *x*). Meanwhile, in the Ω(*x*) region, the similarity function between the pixel value cc of the edge pixel point ξ and the pixel value *f* (ξ) of the pixel point *f* (*x*) in the center region is described by *s* (*f* (ξ), *f* (*x*)). After bifiltering by *f* (*x*), the calculation result is shown by *h* (*x*).

#### Steps of Medical Image Denoising

In order to obtain the denoised medical image, the ultrasonic network signal collected by the ultrasonic imaging system needs to be processed by logarithmic transformation.

(1) Log transforms the ultrasonic network signal collected by the ultrasonic imaging system. If 3D multimodal medical image is directly collected, this step is not required.

(2) Through the image obtained in wavelet decomposition processing step (1), four frequency domains are obtained, which are *LL*^1^, *LH*^1^, *HL*^1^, and *HH*^1^. Continue wavelet decomposition of *LL*^1^ in low-frequency domain and obtain four frequency domains again (18, 19), in order: *LL*^2^, *LL*^2^, *HL*^2^, and *HH*^2^. Perform wavelet decomposition repeatedly until the maximum number of layers is decomposed *J*.

(3) The bottom low-frequency region *LL*^*J*^ is processed by bilateral filtering of Equation (9).

(4) The wavelet coefficients in the high-frequency region (*LH*^*j*^, *HL*^*j*^, *HH*^*j*^, *j* = 1, 2, ..., *J*) of each layer are shrunk by Equation (8) and the SD of noise, image, and noise-free signal in each frequency domain is calculated, respectively.

(5) The inverse wavelet transform is performed on the above parameters to obtain the denoised medical image and complete the denoising.

### DRD U-Net Model Using Deep Reinforcement Learning

This article selects the end-to-end DRD U-Net model to segment the 3D multimodal medical image after denoising. The convolution part of the traditional reinforcement learning model is adjusted to the residual module (8, 20) and multiscale context feature extraction atrous spatial pyramid pooling (ASPP) is introduced into the model, so that images of different sizes can be segmented.

#### Multiscale Context Feature Extraction Module

In order not to increase the parameters rapidly, this article adds extended convolution and introduces two-dimensional convolution to improve the receptive field.

By expanding convolution, the receptive field can be increased when the parameters remain unchanged, so as to ensure that comprehensive context information can be obtained during segmentation (21). It is assumed that ASPP consists of four parallel extended convolutions. At the same time, set the module input as *x* ∈ *R*^4*c*×*h*×*w*^, the expansion rate is set as [3, 5, 7, 9]. Then, Equation (10) can be used to calculate the output of the module.


(10) $$\begin{array}{l} m=concatenate\;(m_{1},m_{2},m_{3},m_{4}) \end{array}$$

where $m_{i}\in R^{c\times h\times w}$, according to the expansion convolution with different expansion rates, a large number of context features are obtained and then a variety of features are spliced to comprehensively extract image multiscale features.

#### Medical Image Segmentation Algorithm of DRD U-Net Model

The model introduces residual network in deep reinforcement learning to make the segmentation result more accurate and improve the training speed of deep reinforcement learning.

##### Residual Structure

The residual module can improve the optimization performance of neural network, make the training process easier, and slow down the degradation speed of deep network. In this article, batch normalization (BN) operation is combined in the residual module and ReLu is selected as the excitation function. At the same time, in order to improve the problem of channel number mismatch during input and output, 1 × 1 convolution is used to speed up the channel number transformation and a large number of parameters are not added to the convolution.

The residual structure of the module actually refers to the introduction of a bypass, so that the input and output data can be added to obtain the deep reinforcement learning model. The introduced bypass is usually called shortcut. Using the residual structure, the convergence speed of learning can be increased. Suppose *b*_1_ is network input, then the output of a residual network unit can be represented by Equation (11).


(11) $$\begin{array}{l} m_{1}=h\;(b_{1})+I\;(b_{1},w_{1}) \end{array}$$

where the function to be fitted in the network is described by *h* (·), the convolution calculation is described by *I* (*b*_1_, *w*_1_), the weight parameter in the network is *w*_1_, and the first layer network output is *m*_1_. If *h* (·) belongs to identity mapping, *h* (*b*_1_) = *b*_1_. Therefore, the network output *m*_1_ of the first floor residual structure can be calculated by Equation (12).


(12) $$\begin{array}{l} m_{1}=b_{1}+1=b_{1}+I\;(b_{1},w_{1}) \end{array}$$

After the initial data of the input image and the output data after convolution calculation are linearly added, it is all the outputs of the residual unit. Therefore, one addition algorithm can be used to describe all the network outputs. If there are *N* residual structure network modules in the whole network, the overall network output can be calculated by Equation (13).


(13) $$\begin{array}{l} b_{N}=b_{n}+\sum_{i=n}^{N-1}F\;\left(b_{i},W_{i}\right) \end{array}$$

##### Medical Image Segmentation of Joint Residual Structure and DRD U-Net Convolution

For 3D multimodal medical image, the edge of medical image has high complexity, so it is usually difficult to effectively segment the lesion area from the normal area. Therefore, by adding the residual structure, the initial output characteristics of the image are retained to prevent the edge data around the tissue from being damaged during segmentation. At the same time, the local data in multimodal medical image can be fully combined during training.

In this article, the convolution layer in the residual module is set as 3D convolution form, a multiscale context feature extraction module is added, and the BN layer is adjusted to GN layer. According to the convolution kernel size, construct the convolution layer of 3 × 3 × 3. Then, it is introduced into the GN layer to improve the convergence ability of the network and then input to the non-linear activation function layer. Next, it is input to a 3 × 3 × 3 convolution kernel again. In order to accurately extract context features, two 3D convolution layers are used to increase the depth of the network. On this basis, the computational complexity can be effectively improved. Using the introduced residual structure, the feature extraction performance of the network can be improved.

#### Medical Image Segmentation Algorithm for Three-Dimensional Multimodal

With its powerful feature representation ability, deep reinforcement learning can accurately segment multidimensional and multimodal medical images. The 3D multimodal medical image segmentation algorithm based on deep reinforcement learning is as follows:


(14) $$\begin{array}{l} W=\frac{B}{H}\times \frac{u\cap v}{u\cup v} \end{array}$$

where *B* refers to obtain the pixel value of the target of interest in the medical image, *H* refers to the total pixel value in the medical image obtained, *v* refers to the region where interested target locates, and *v* refers to the region where interested denoised target locates.

Negative rewards often occur in the process of deep reinforcement learning. In order to solve these problems, the reward method from the starting point of intermediate difficulty is used to calculate from the intermediate process. The reward calculation of medical image segmentation is as follows:


(15) $$\begin{array}{l} f\;(l_{t},m_{t})=sign\;(W\;(s_{t+1},n)-W\;(s_{t},n)) \end{array}$$

where after the best segmentation *m*_*t*_, get the next monitoring region *s*_*t*+1_, and predict the reward according to the segmented *W*. If the image segmentation at time *t*+1 is greater than the image segmentation at time *t*, a reward is given. Otherwise, a penalty is given. Through the reward and penalty mechanism, the incorrect segmentation of medical images is minimized. The image segmentation at time *t* can be expressed by the following Equation:


(16) $$\begin{array}{l} Q=\overset{t_{\max}}{\underset{t=1}{\sum }}\kappa^{t-1}f_{t} \end{array}$$

where κ ∈ [0, 1] represents the discount factor. The greater its value, the greater the total reward.

To prevent local minima, use χ to mean the accurate qualitative strategy, χ begins from 0.9 and decreases by 0.1 each time till χ remains at 0.1.


(17) $$\begin{array}{l} m_{t}=\{\begin{array}{c} \text{Re-segmentation},\begin{array}{c} \text{ Under }\chi \;\text{probability} \end{array}\\ P\;(l_{t},m_{t}),\;Others \end{array} \end{array}$$

**Figure 1:**
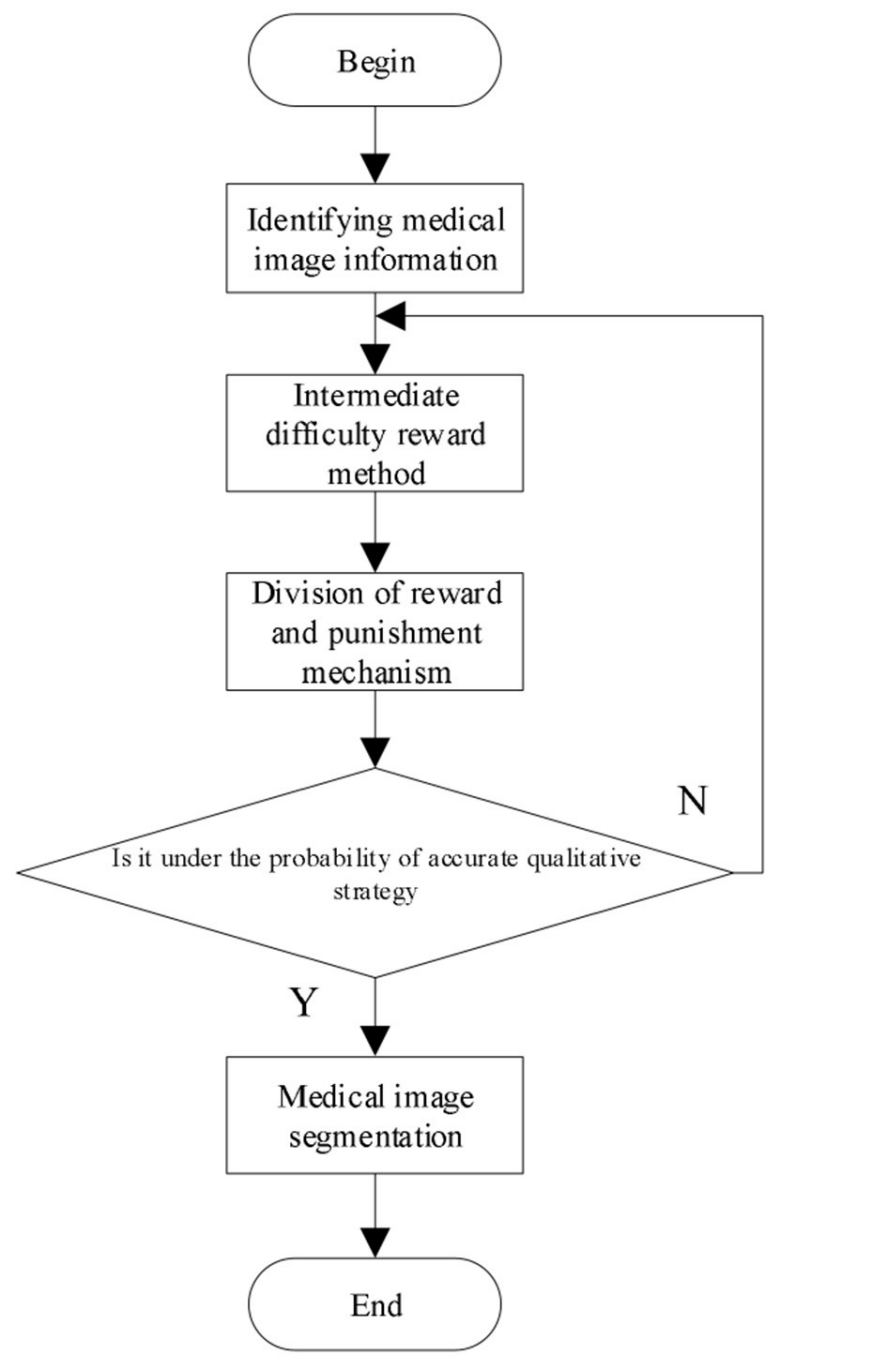
Process of three-dimensional (3D) multimodal medical image segmentation.

Through the above calculation process, the 3D multimodal medical image segmentation is realized and the calculation process is shown in Figure 1.

## Experimental Results and Analysis

### Data Set

In the experiment, PyTorch is used to build the structural framework of deep reinforcement learning and python language is used to complete the programming of medical image segmentation algorithm.

In order to verify the effectiveness of 3D multimodal medical image segmentation algorithm based on deep reinforcement learning, the algorithm is verified by experiments. The LIDC-IDRI data set, the Segmentation in Chest Radiographs (SCR) data set, and the DeepLesion data set are selected as the experimental data set of this article. The LIDC-IDRI data set: This dataset includes diagnostic and screening chest CT scans for lung cancer. A total of 1,018 study examples are included in the data set, with annotated lesions indicated. The SCR data set: This data set is a public database containing 247 chest images with segmentation of each image. The DeepLesion data set: This data set is an open dataset of CT images, including 32,735 CT images and lesion information from 4,427 patients, of which brain images and lung images are selected for this experiment. From the above three datasets, 5,000 medical images were selected for experimental analysis. The image sizes in the medical data were adjusted to 84 × 84 and the resolution of the images was 0.84 × 0.84 × 3 mm. These experimental data were randomly sorted and 4,000 images were selected as the training data set and 1,000 images were selected as the test data set for the experiments in this article.

### Evaluation Criteria

1 Structural similarity: Medical image has a good structure and the pixels of image have many important correlations. The calculation procedure is shown in Equation (18).


(18) $$\begin{array}{l} SSIM\;(X,Y)={\left[l\;(X,Y)\right]}^{\alpha }{\left[c\;(X,Y)\right]}^{\beta }{\left[s\;(X,Y)\right]}^{\gamma } \end{array}$$

where brightness comparison is described by *l* (*X, Y*), contrast comparison is described by *c* (*X, Y*), image structure comparison is described by *s* (*X, Y*), through parameters α, β, and γ, adjust the relative importance of the above three components. The greater the structural similarity, the better the segmentation effect.

2 Signal-to-noise ratio: The ratio between image information and noise is the SNR. Through the analysis of SNR, we can get the noise situation after image segmentation. When the SNR is higher, it means that the segmentation effect is better.

3 Training loss: The loss is a numerical value indicating the accuracy of image segmentation. If the segmented image is the same as the actual image, the loss is zero.

4 Relative overlap: The segmentation performance verification criteria is defined by the Equation (19) and the relative overlap is shown in Equation (19).


(19) $$\begin{array}{l} R\text{\_}overlap=\frac{A\;(T\cap S)}{A\;(T\cup S)} \end{array}$$

where the overall target area is described by *A* (*T*), the size of the segmented target area is described by *A* (*S*), and the higher the *R*_*overlap*, the more accurate the segmentation.

5 Accuracy: Accuracy analysis is performed on the test data set, when the greater the accuracy, the more accurate the segmented image is, using the Equation (20) to calculate the accuracy.


(20) $$\begin{array}{l} P=\frac{A\;(T\cap S)}{A\;(S)} \end{array}$$

## Results and Discussion

In order to verify the effectiveness of the 3D multimodal medical image segmentation algorithm based on deep reinforcement learning, the proposed algorithm is validated by simulation experiments.

**Figure 2:**
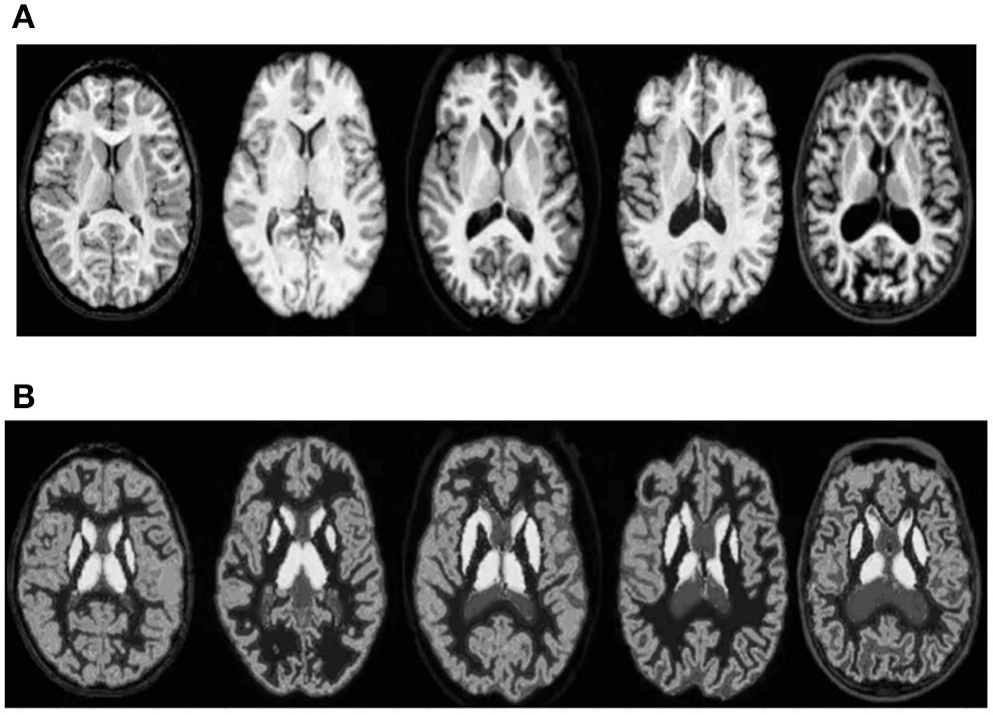
Comparison of the effect of brain image segmentation before and after. **(A)** Presegmentation brain images. **(B)** Brain images postsegmentation by proposed algorithm.

**Figure 3:**
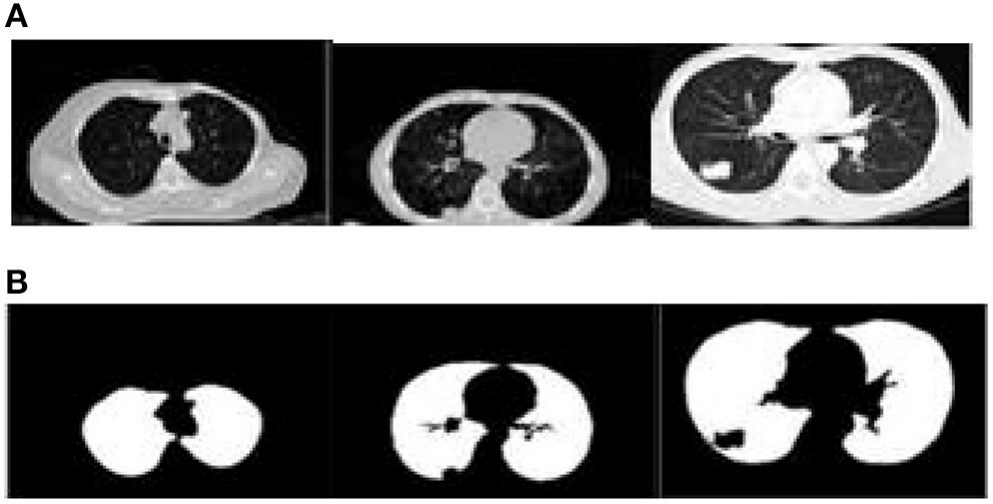
Comparison of the effect of lung image segmentation before and after. **(A)** Presegmentation lung images. **(B)** Lung images postsegmentation by proposed algorithm.

The proposed algorithm is used to segment the 3D multimodal medical images in the test set. Five brain images in the DeepLesion data set and three lung images in the SCR data set are selected for this study and the comparison of the effect pre- and postsegmentation is shown in Figures 2, 3.

According to Figures 2, 3, the application of the segmentation proposed algorithm can effectively achieve medical image segmentation. The image edge is clear for postsegmentation, the noise points can be effectively removed, and the definition is high.

In order to further verify the performance of the segmentation algorithm, the structural similarity, SNR, training loss, relative overlap, and accuracy are selected to verify the actual performance of the algorithm.

**Figure 4:**
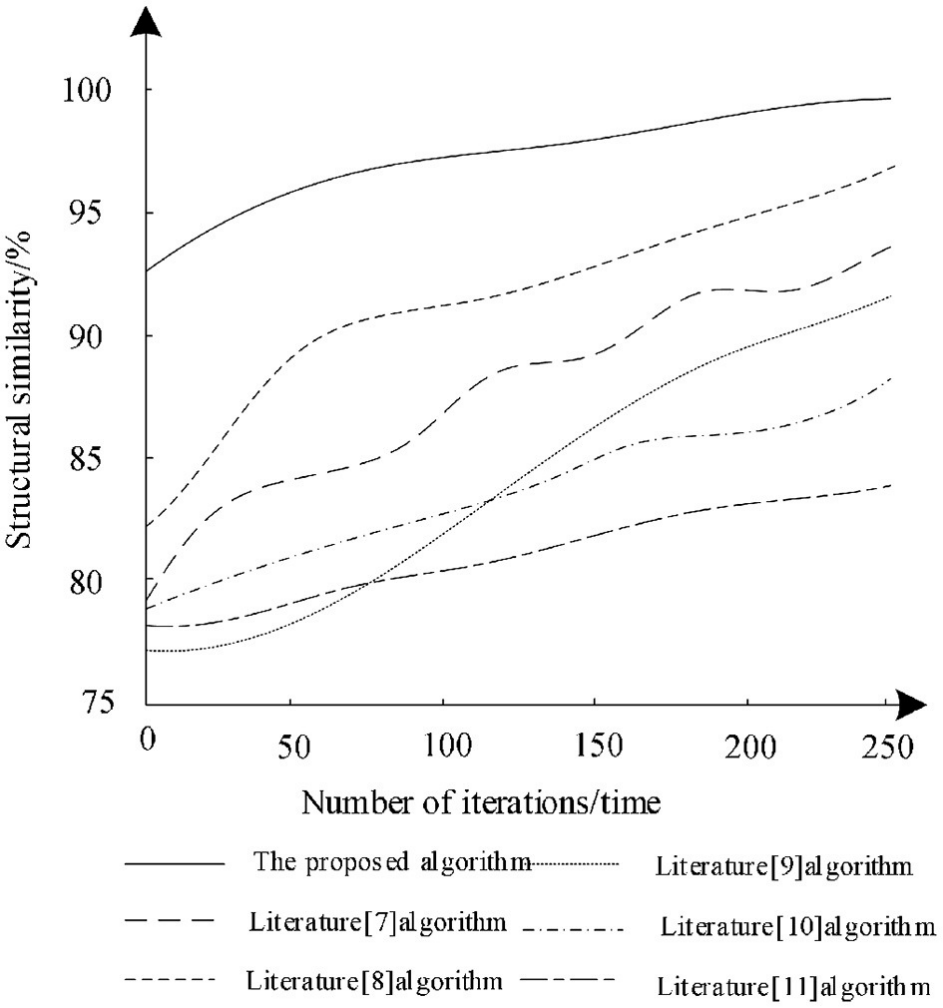
Comparison of structural similarity of different algorithms.

The training data set was trained with different iterations to analyze the structural similarity of the different algorithms after training and the analysis results are shown in Figure 4.

According to Figure 4, with the increase of the number of iterations, the structural similarity of different algorithms increases, which shows that the training of each algorithm can optimize the algorithm. Among them, the structural similarity of the algorithm in literature (11) remains the lowest in the last iteration. Therefore, the segmentation performance of the algorithm is poor, while the structural similarity of the algorithm in literature (8) increases rapidly during the iteration and finally reaches 96%. This shows that the algorithm can effectively ensure the structural similarity of the image during segmentation, but the algorithm is still lower than the proposed algorithm. When the number of training iterations is 250, the structural similarity reaches 98% and each iteration has the highest structural similarity. Therefore, compared with other algorithms, the segmentation training effect of proposed algorithm is better. The proposed algorithm combined with bilateral filter to filter the low-frequency wavelet coefficients and uses deep reinforcement learning to segment the medical image, which can enhance the structural similarity of image.

**Figure 5:**
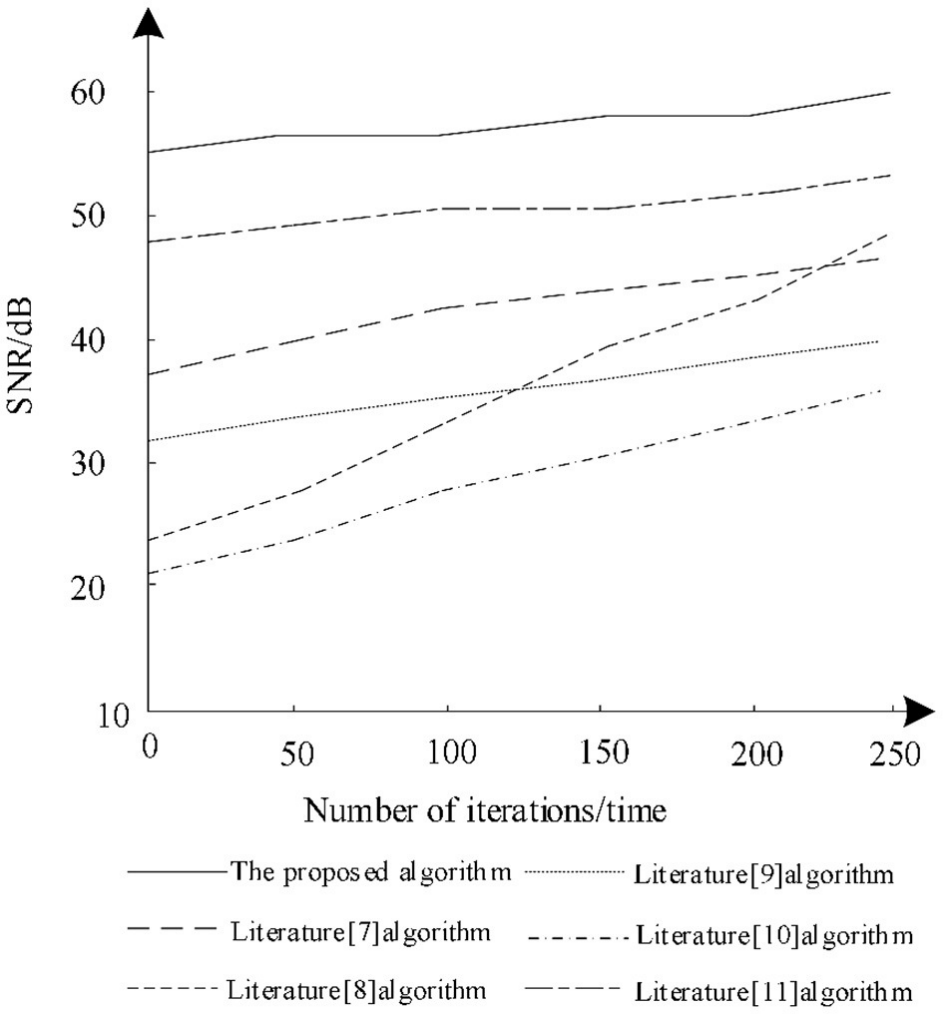
Comparison of the signal-to-noise ratio (SNR) variation of different algorithms.

The ratio between image information and noise is the SNR. Through the analysis of SNR, the noise content after image segmentation can be obtained. The higher the SNR, the better the segmentation effect, so as to analyze the changes of the SNR of different algorithms after training. The results of the analysis are shown in Figure 5.

It is seen from Figure 5 that when the number of iterations increases gradually, the SNR increases postsegmentation by different algorithms. Among them, the SNR of the algorithm in literature (8) increases greatly, gradually from 24 to 47 dB, but the SNR of the algorithm is still lower than that in literature (11) and the proposed algorithm. The SNR of the algorithm in literature (10) remains the lowest during iteration and there is no significant increase and the maximum SNR is only 33 dB, which shows that the algorithm has low noise reduction ability during training. The proposed algorithm always has a high SNR during training and is very stable. Under different iterations, the SNR is always maintained between 55 and 60 dB. Therefore, using proposed algorithm for segmentation can effectively reduce image noise.

**Figure 6:**
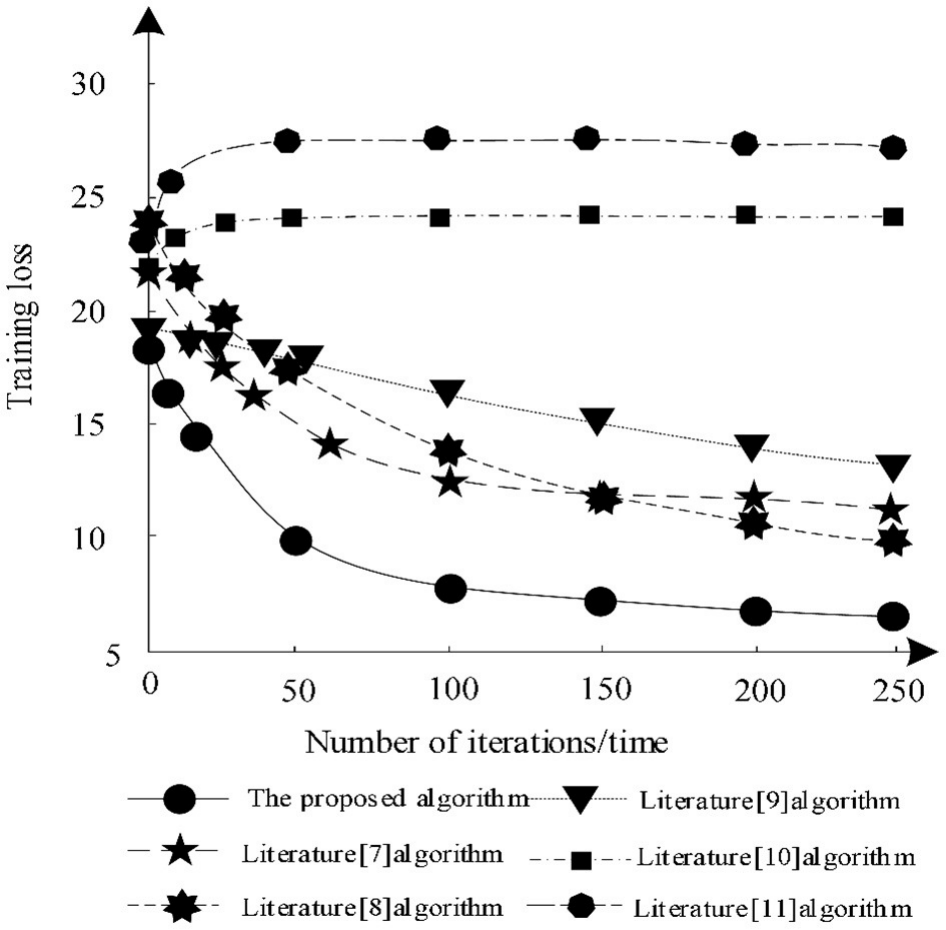
Training loss of different algorithms.

The training loss of each algorithm segmentation under different training times is analyzed and the analysis results are shown in Figure 6.

According to Figure 6, when the number of iterations increases, the training losses of algorithm in literature (7), algorithm in literature (8), algorithm in literature (9), and algorithm in this article gradually decrease, while the training losses of algorithm in literature (10) and algorithm in literature (11) show an upward trend. Among them, the training loss of the algorithm in literature (11) is always the highest, indicating that the algorithm always has a large loss during segmentation training and cannot guarantee the integrity of the image. The training loss of the proposed algorithm decreases gradually in the iterative process, which shows that after training, the proposed algorithm can make the image segmentation more complete and ensure the effectiveness of segmentation. Because proposed algorithm uses DRD U-Net model, the model introduces residual network in deep reinforcement learning, which makes the segmentation result more accurate and the training loss less.

The relative overlap analysis is performed on the test image data set by comparing the proposed algorithm with those of literature (7), literature (8), literature (9), literature (10), and literature (11). The experimental results are shown in Table 1.

**Table 1:** Comparison of the relative overlap of different algorithms.

**Algorithms**	**Relative overlap**		
	**LIDC-IDRI**	**SCR**	**DeepLesion**
The proposed	0.95	0.96	0.95
Literature (7)	0.89	0.80	0.84
Literature (8)	0.85	0.81	0.85
Literature (9)	0.87	0.81	0.81
Literature (10)	0.83	0.80	0.80
Literature (11)	0.85	0.84	0.87

As can be seen from Table 1, the relative overlap of each algorithm reaches more than 80%. Among them, there are small differences in the relative overlap of the algorithms under the three data set tests of the LIDC-IDRI, the SCR, and the DeepLesion, but in terms of numerical size comparison, the proposed algorithm always maintains a high relative overlap of up to 95%, which is much higher than other algorithms in the literature, indicating that the proposed algorithm can achieve a more complete segmentation. Because the proposed algorithm uses deep reinforcement learning methods, with its powerful feature representation capability, it can accurately segment multidimensional multimodal medical images through its reward and punishment mechanism, thus improving the relative overlap of segmentation results.

**Figure 7:**
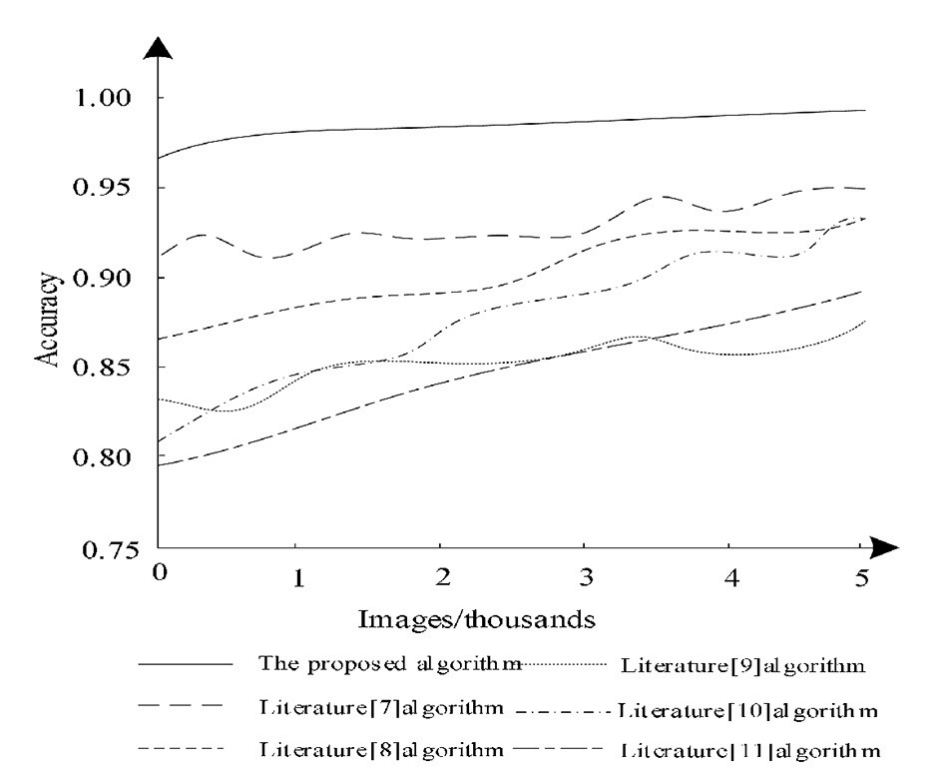
Comparison of segmentation accuracy of different algorithms.

The accuracy analysis was performed on the test dataset, when the larger the accuracy value, the better the image segmentation effect. The test images were analyzed using Equation (20) and the segmentation accuracy capabilities of different algorithms were compared and the analysis results are shown in Figure 7.

According to Figure 7, it can be seen that when the number of images increases, the segmentation accuracy of different algorithms changes. Among them, the algorithm of literature (11) has a large increase, but its segmentation accuracy always remains low and the segmentation accuracy of literature (7) is higher compared to other algorithms in literature, but the highest does not exceed 95%, whereas the proposed algorithm always maintains the highest accuracy and is 95% when performing segmentation. Therefore, it is clear that the proposed algorithm can effectively guarantee the accuracy of image segmentation. Because the proposed algorithm uses deep reinforcement learning method, proposed algorithm used the framework of deep learning and reinforcement learning thinking, which can segment medical images accurately.

## Conclusion

This article proposed the 3D multimodal medical image segmentation algorithm based on deep reinforcement learning, so as to realize the segmentation of 3D multimodal medical image through deep reinforcement learning training and verify the actual performance of the algorithm by experiments. Experiments show that the algorithm in this article has high relative overlap, structural similarity, and quality factors and has strong image segmentation ability and high definition. It can effectively reduce image noise and has high application effect. In the future studies, we can continue to optimize according to the existing research theory, continuously strengthen the segmentation ability of medical images, and realize the effective segmentation of various types of medical images.

## Data Availability Statement

The original contributions presented in the study are included in the article/supplementary material, further inquiries can be directed to the corresponding author.

## Author Contributions

WG and XL: conception and writing. YW: investigation and methodology. YC: data and validation. All authors contributed to the article and approved the submitted version.

## Funding

This study was supported by the Natural Science Foundation of Heilongjiang Province of China under grant number LH2021F039.

## Conflict of Interest

The authors declare that the research was conducted in the absence of any commercial or financial relationships that could be construed as a potential conflict of interest.

## Publisher's Note

All claims expressed in this article are solely those of the authors and do not necessarily represent those of their affiliated organizations, or those of the publisher, the editors and the reviewers. Any product that may be evaluated in this article, or claim that may be made by its manufacturer, is not guaranteed or endorsed by the publisher.
